# A Data Fusion Orientation Algorithm Based on the Weighted Histogram Statistics for Vector Hydrophone Vertical Array

**DOI:** 10.3390/s20195619

**Published:** 2020-10-01

**Authors:** Yan Liang, Zhou Meng, Yu Chen, Yichi Zhang, Mingyang Wang, Xin Zhou

**Affiliations:** 1 College of Advanced Interdisciplinary Studies, National University of Defense Technology, Changsha 410073, China; liangyan18@nudt.edu.cn (Y.L.); gfkdzyc@163.com (Y.Z.); wmypis@126.com (M.W.); 15580924436@163.com (X.Z.); 2 College of Meteorology and Oceanography, National University of Defense Technology, Changsha 410073, China; zhoumeng6806@163.com; 3 Academy of Artillery and Air Defense, Nanjing 210000, China

**Keywords:** direction-of-arrival (DOA), vector hydrophone vertical array (VHVA), data fusion, weighted histogram statistics, high-resolution, sub-band, deep-sea target detection

## Abstract

In this paper, we propose a data fusion algorithm based on the weighted histogram statistics (DF-WHS) to improve the performance of direction-of-arrival (DOA) estimation for the vector hydrophone vertical array (VHVA). The processing frequency band is firstly divided into multiple sub-bands, and the high-resolution multiple signal classification (MUSIC) algorithm is applied to estimate the azimuth of each sub-band for each vector hydrophone. Then, the weighted least square (WLS) data fusion technique is used to fuse the sub-band estimation results of multiple sensors. Finally, the weighted histogram statistics method is employed to obtain the synthesis results in the frequency domain. We carried out a simulation and sea trial of the 16-element VHVA to evaluate the performance of the proposed algorithm. Compared to several traditional processing algorithms, the beam width of the proposed approach is significantly narrower, the side lobes are considerably lower, and the mean square error (MSE) is effectively smaller. In addition, the DF-WHS method is more suitable to accurately estimate the target azimuth with a low signal-to-noise ratio (SNR) because the noise sub-band is suppressed in the weighted histogram statistics step. The DF-WHS method in this article provides a new approach to improve the performance of deep-sea target detection for the VHVA.

## 1. Introduction

The vector hydrophone is a new type of underwater acoustic measurement equipment composed of a sound pressure hydrophone and a particle velocity hydrophone. It can simultaneously measure underwater sound pressure and the scalar and orthogonal particle velocity vector of the sound field. Some types of vector hydrophones measure the acceleration of the sound field directly and then convert it into a velocity vector. Vector hydrophones are widely used in underwater acoustic detection, communication and marine environment monitoring, and so on. In comparison with traditional omnidirectional sensors, a single vector hydrophone can estimate the target azimuth due to its intrinsic dipole directivity. In the past few decades, a large number of reports have been made regarding target orientation algorithms for a vector hydrophone. Gordienko et al. [1] adopted the sound energy flow algorithm to obtain the target azimuth and performed direction-finding accuracy analysis. Nehorai et al. [2,3] systematically proposed methods for the first time in which the acoustic energy flow direction-finding algorithm and eigenvector algorithm for a vector sensor were applied. The direction-of-arrival (DOA) estimation performance of these methods was proven to be close to the Cramér–Rao bound (CRB). Later, Felisberto et al. [4] presented a means to estimate the azimuths of multiple sources with non-overlapping bands based on the inner product between the sampled sound field and the different velocity orthogonal components.

However, due to the complex environments of the ocean, the orientation accuracy for a single vector sensor is not high enough and the resolution is insufficient in the case of multiple targets. To improve the target detection performance, the line array composed with multiple vector hydrophones is often adopted, and traditional array signal processing algorithms commonly used in the pressure hydrophone array have also been introduced. Hawkes [5] introduced conventional beamforming (CBF) and minimum variance distortionless response (MVDR) adaptive beamforming technology into vector array signal processing and achieved better performance than that of the corresponding scalar array. Reference [6] described the beamspace extension of the channel cross-correlation method and two other eigen-vector-based approaches to perform DOA estimation. To further improve the accuracy of azimuth estimation, researchers introduced high-resolution algorithms into vector sensor array processing. K. T. Wong [7,8] first proposed the high-resolution multiple signal classification (MUSIC) algorithm designed for vector sensors to solve the problem of the accurate orientation of targets. Reference [9] introduced the estimation of signal parameters via rotational invariance techniques (ESPRIT) into the vector array. For different array shapes, they proposed some improved ESPRIT algorithms, which are not affected by array position errors and have strong adaptability [10,11]. Yu et al. [12] proposed a method that can simultaneously estimate two-dimensional DOAs and frequency. References [13,14] enriched ESPRIT technology and applied it in practice. Researchers have also adopted a vector sensor array to the experiment for DOA estimation and verified its availability [15,16,17,18].

The orientation algorithms mentioned above have proved to be suitable for azimuth estimation for the vector hydrophone horizontal array. However, it is difficult to significantly improve the azimuth estimation accuracy when applying these methods to the vector hydrophone vertical array. According to the bridge product theorem, the directivity of the vector sensor array in any direction can be expressed as the product between the directivity of the non-directional pressure array and the dipole directivity of a single vector sensor [19]. In addition, the directivity of the pressure vertical array is only related to the elevation angle and is not sensitive to the changes of azimuth [20]. Therefore, compared with the DOA estimation results of a single sensor, it is hard to obtain better results when applying existing array signal processing algorithms to the vector hydrophone vertical array.

To solve this problem, researchers introduced multi-sensor data fusion technology. Data fusion is defined as a technical method that combines data from multiple sensors with information from related databases to achieve higher accuracy and more specific inference [21,22]. Multi-sensor data fusion technology was initially applied in the military industry, such as in automated target recognition, digital signal processing, artificial intelligence, and other technical methods [23]. In the middle of the 20th century, this technology was introduced into the underwater acoustic field, and was mainly used for underwater detection, target tracking, target recognition, etc. In [24], a method combining least mean square estimation and wavelet denoising was proposed. Zhang et al. [25] made full use of the multi-input of each vector hydrophone and designed the weights according to the principle of the minimum mean square error, aiming at improving the fusion precision. Multi-sensor fusion technology has also been applied to autonomous navigation and underwater localization. Li et al. [26] fused the data from bow and side arrays to improve detection performance, and the simulation results verify the approach’s effectiveness, particularly with a low signal-to-noise ratio (SNR). Dos Santos et al. [27] combined deep neural networks and adaptive Monte Carlo localization to achieve underwater localization by fusing data from multiple image domains. Tollefsen et al. [28,29] further enriched the underwater fusion technology and considered approaches to synthesize information from multiple arrays for underwater source localization.

In this paper, we propose a data fusion orientation method based on the weighted histogram statistics (DF-WHS) to take full advantage of multiple outputs for the vector hydrophone vertical array. A simulation and sea trial of the 16-element vector hydrophone vertical array in the deep ocean were conducted. Both results indicate that the DF-WHS algorithm can suppress noise effectively and achieve more stable and accurate DOA estimation results than other conventional methods.

This paper is organized as follows. In Section 2, we introduce the basic theory of the vector hydrophone and the methods involved. A simulation is carried out to evaluate the performance of the algorithm proposed under different situations in Section 3. Section 4 analyzes the sea trial results. Section 5 summarizes the conclusions.

## 2. Basic Theory

### 2.1. Principle of the Vector Hydrophone

As mentioned in the introduction, vector hydrophones can simultaneously collect sound pressure and orthogonal particle velocity information. There are two common types of vector hydrophones, namely, the piezoelectric and the optical fiber type. The optical fiber vector hydrophone has the advantages of high sensitivity, large dynamic range and strong anti-electromagnetic interference ability, etc. Therefore, here, we introduce the principle of the optical fiber vector hydrophone and use it in the experiment.

**Figure 1:**
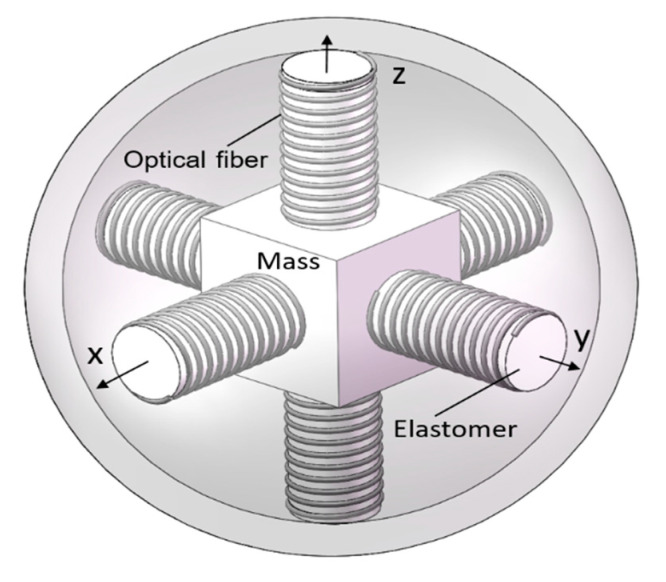
The composition of the accelerometer.

In practical application, the co-vibration type optical fiber vector hydrophone is common. It adopts a rigid thin-shell structure, and its interior consists of an optical fiber pressure hydrophone and an optical fiber three-dimensional orthogonal accelerometer that is regarded as the core component. As shown in Figure 1, the accelerometer is made up of a mass block M and six elastomers. The elastomer is tightly wound by a sensing optical fiber, and the two opposite elastomers form the two arms of each Michelson interferometer.

When the water flow pushes the spherical shell, the shell begins to move. Under the action of inertial force, the mass block M begins to vibrate along three axes—x, y, and z—and the six elastomers around the mass block are subsequently compressed or stretched along the axial direction. As a result, the length of the optical fiber wound on the elastomer changes, and then interference effect occurs, and a phase difference is formed in each axial direction. Finally, the phase difference is converted into acceleration through the photoelectric detector, and thus, the acceleration information in three directions of the vector hydrophone is obtained [30].

**Figure 2:**
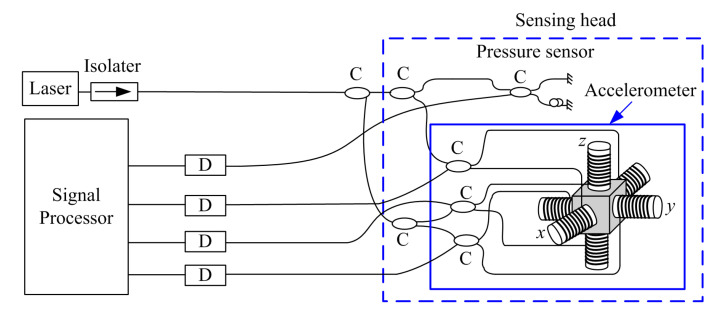
Structure of optical fiber vector hydrophone.

The above is a description of the principle of the accelerometer. For the pressure sensor, the sensing head receives underwater pressure vibration and converts it into optical fiber strain changes. After the coupler output, the interference effect is produced, and the phase difference is formed. Figure 2 is the optical path diagram of the optical fiber vector hydrophone.

The optical fiber vector hydrophone consists of three parts, that is, a light source, a sensing head, and a module for signal detection and demodulation. The sensing head is formed of an optical pressure sensor and a three-dimensional orthogonal optical fiber accelerometer [31]. 

The light emitted from the light source passes through the coupler (C) and enters the optical fiber pressure hydrophone and the three axes of the accelerometer. 

The returned light is converted into electrical signals by the photodetector (D) and then enters the signal processor. Then, acceleration is transformed into particle velocity in subsequent processing. According to the kinematics theory, the relationship between acceleration and velocity can be described as follows:

(1) $$v=v_{0}+\int_{t_{1}}^{t_{2}}a\cdot dt$$

where the $v_{0}$ represents the initial velocity and a is the acceleration. $t_{1}$ and $t_{2}$ stand for the upper and lower limits of the integral, respectively. 

The optical isolater is set at the rear of the laser to prevent the adverse effects of the backward transmitted light on the light source and the optical system. 

**Figure 3:**
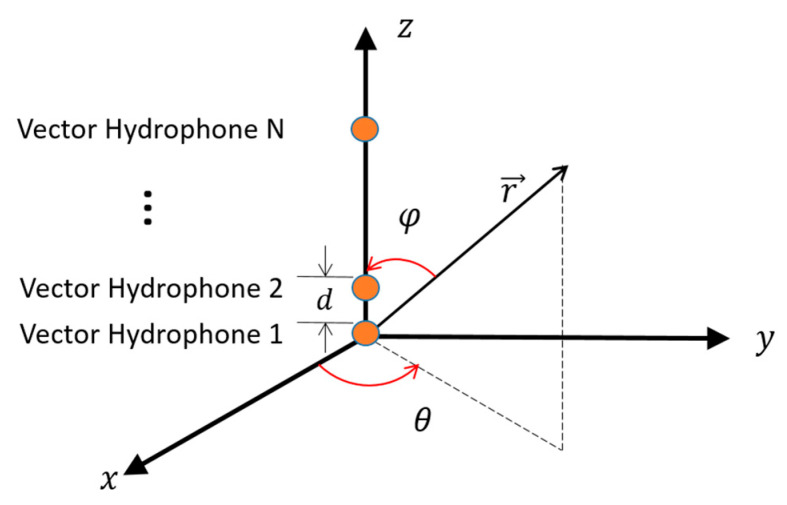
Schematic diagram of vector hydrophone vertical array.

The vector hydrophone vertical array is a series of such vector hydrophones connected at different depths in the vertical direction, of which the schematic diagram is shown in Figure 3. θ and φ denote the azimuth and elevation angle of the source, respectively. The element interval is d.

In our paper, the vector hydrophones are only used to collect signal radiated from the acoustic source. The target azimuth is calculated by the subsequent signal processing algorithm. 

### 2.2. Receiving Model of a Single Vector Hydrophone

Compared with the traditional scalar hydrophone, the vector hydrophone can obtain not only the scalar sound pressure information p, but also the orthogonal vector velocity information v. Under the assumption of the ideal fluid medium with uniform and static characteristics, the relationship between the particle velocity and the sound pressure satisfies the Euler equation as:

(2) $$v=-\frac{1}{\rho }\int \nabla p\cdot dt$$

where t represents the time, ρ stands for the medium density, and the symbol ∇ is the Hamiltonian operator.

Under the far-field condition, the acoustic signal in space can be approximately regarded as a plane wave:

(3) $$p\left(r,t\right)=Ae^{j\left(\omega t-kr\right)}$$

where A is the amplitude, ω represents the angular frequency, k=ω/c represents the wave number, and r is the position vector.

Substituting Equation (3) into Equation (2), the particle velocity can be expressed as:

(4) $$v\left(r,t\right)=\frac{A}{\rho c}e^{j\left(\omega t-kr\right)}=\frac{p\left(r,t\right)}{\rho c}$$

It can be seen from Equation (4) that the sound pressure and particle velocity in the plane wave model have the same phase, and only differ by a coefficient-acoustic impedance ρc.

**Figure 4:**
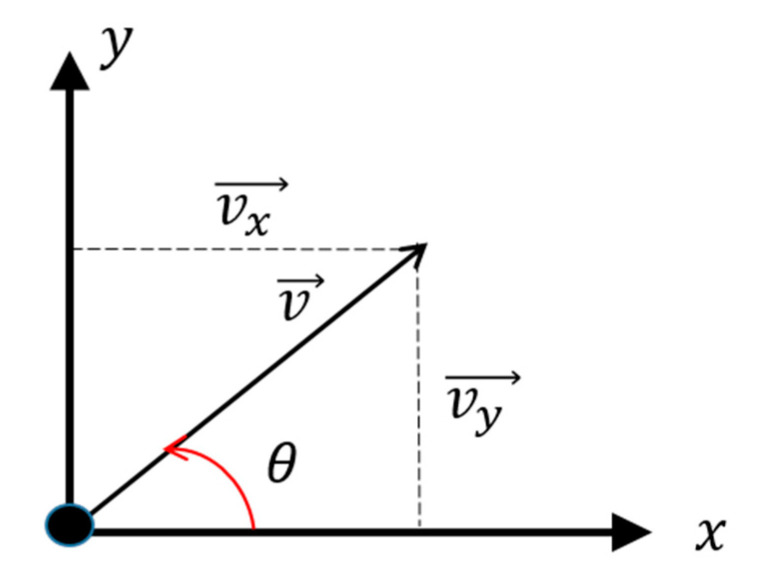
Schematic diagram of the particle velocity and two-dimensional orthogonal components.

For the convenience of discussion, the acoustic impedance ρc is often neglected. Generally, v(t) contains three orthogonal components.

(5) $$\{\begin{array}{c} v_{x}\left(t\right)=p\left(t\right)\cdot \sin\varphi \cos\theta \\ v_{sy}\left(t\right)=p\left(t\right)\cdot \sin\varphi \sin\theta \\ v_{sz}\left(t\right)=p\left(t\right)\cdot \cos\varphi \end{array}$$

where φ represents the elevation angle and ranges from 0 to π. θ is the azimuth angle and ranges from 0 to 2π. It can be seen that the azimuth θ is only related to the X and Y axis components, not to the Z axis component. Therefore, we consider the particle velocity under two-dimensional conditions. A schematic diagram of the particle velocity and two-dimensional orthogonal components is presented in Figure 4.

The sound pressure and two-dimensional velocity components received by a vector hydrophone can be expressed as:

(6) $$\{\begin{array}{c} p_{s}\left(t\right)=p\left(t\right)\\ v_{sx}\left(t\right)=p\left(t\right)\cdot \cos\theta \\ v_{sy}\left(t\right)=p\left(t\right)\cdot \sin\theta \end{array}$$

Generally speaking, a single vector hydrophone can be equivalent to a small three-element array. Assuming that there are k far-field narrowband signals incident on the array with the azimuth angle vector $\theta_{k}=\left[\theta_{1},\theta_{2},\cdots \theta_{l},\cdots \theta_{k}\right]$, the signal received by the equivalent array can be written as:

(7) X(n)=A(Θ)S(n)+N(n)

where $A\left(\Theta \right)=\left[a\left(\theta_{1}\right)\;a\left(\theta_{2}\right)\;\cdots a\left(\theta_{k}\right)\right]$ is the matrix of the array manifold, $a\left(\theta_{l}\right)={\left[1\cos\theta_{l}\sin\theta_{l}\right]}^{T}$ is the array manifold vector of the l-th narrowband signal, and ${\left[\;\right]}^{T}$ is the transposition operator. $S\left(n\right)=\left[s_{1}\left(n\right)\;s_{2}\left(n\right)\;\cdots s_{k}\left(n\right)\right]$ is the k×1 dimension incident signal vector and N(n) denotes the 3×1 dimension noise vector.

### 2.3. Horizontal Directivity Index of the Vector Hydrophone Vertical Array

Assume there is a vertical line array composed of N vector hydrophones and the element interval is d. As shown in Figure 3, the vertical array is placed along the z−axis and the reference element is at the origin of the coordinate. Suppose that the sound source is located in the far-field and radiates plane waves. The pressure signal received by the i-th element can be expressed as:

(8) $$p_{i}\left(t\right)=e^{j\left(\omega t-kr_{i}\right)}\;i=1,2,\ldots N$$

where $r_{i}$ denotes the distance from the sound source to the i-th element, which can be expressed as:

(9) $$r_{i}=R-d\cos\varphi \;i=1,2,\ldots N$$

where R represents the distance from the sound source to the reference element.

Further, the directivity function for the pressure hydrophone vertical array can be written as [20]:

(10) $$B_{p}=\left|\frac{\sin[N\pi d(\cos\varphi -\cos\varphi_{0})/\lambda ]}{N\sin[\pi d(\cos\varphi -\cos\varphi_{0})/\lambda ]}\right|$$

where $\varphi_{0}$ represents the steering elevation angle and λ is the wavelength of the sound wave.

From Equation (10), we can see that the directivity function of the pressure sensor vertical array is not sensitive to the variation of azimuth. According to the pattern product theorem [32], the directivity of the vector hydrophone vertical array can be regarded as the product of the directivity of a single vector hydrophone and that of the corresponding pressure hydrophone vertical array. Hence, the horizontal directivity for the vector hydrophone vertical array is merely contributed by a single vector element and it is difficult to achieve significantly improved azimuth estimation performance using conventional array signal processing methods.

### 2.4. Cross-Spectrum Sound Intensity Method

In the case of an isotropic noise field, the sound intensity estimation is the maximum likelihood detection of the vector hydrophone [33]. Generally, the product of sound pressure and particle velocity is defined as sound intensity, which can be written as:

(11) I(t)=p(t)⋅v(t)

The cross-spectrum sound intensity method involves processing the product of sound pressure and velocity in the frequency domain. It can be seen that the azimuth is only related to the X and Y axis components, not to the Z axis component. Under two-dimensional conditions, the sound intensity is expressed as:

(12) $$I_{i}\left(\omega \right)=\bar{P\left(\omega \right)\cdot V_{i}{}^{*}\left(\omega \right)}i=x,y$$

where P(ω) and $V_{i}\left(\omega \right)$ are Fourier transforms of pressure p(t) and particle velocity component $v_{i}\left(t\right)$, respectively. ω is the angular frequency of received signal, and the symbol ∗ denotes the conjugate operator. $\bar{\left(\;\right)}$ means to average the cross-spectrum sound intensity.

According to the analysis above, the sound pressure and velocity have the same phase in the plane wave sound field. After the Fourier transform, the signal energy is concentrated on the real part of the cross-spectrum output, namely:

(13) IRi(ω)=Re[Ii(ω)] i=x,y

The azimuth of the incident signal at angular frequency ω can be expressed as:

(14) $$\theta \left(\omega \right)=\arctan\frac{I_{Ry}\left(\omega \right)}{I_{Rx}\left(\omega \right)}$$

Equation (14) can be used to calculate the azimuth at each frequency. For the broadband signal, the conventional histogram statistics method is often used to obtain the statistical azimuth results of multiple frequency points. Ultimately, the angle corresponding to the maximum statistical value is the target azimuth.

### 2.5. MUSIC Algorithm of a Single Vector Hydrophone

Multiple signal classification (MUSIC) is a widely used high-resolution orientation algorithm. Suppose that the signal is uncorrelated to the noise, the covariance matrix of the received data is written as:

(15) $$R=E\left\{XX^{H}\right\}=A\left(\Theta \right)R_{s}A^{H}\left(\Theta \right)+R_{n}$$

where $R_{s}$ and $R_{n}$ denote the covariance matrix of signal and noise, respectively. The superscript H is the conjugation operator.

Decomposing the eigenvalue of Equation (15), the covariance matrix of the received data can be rewritten as:

(16) $$R=U_{s}\Sigma_{s}U_{s}{}^{H}+U_{n}\Sigma_{n}U_{n}{}^{H}$$

where $U_{s}$ is the subspace of the eigenvectors corresponding to the k larger eigenvalues of R, i.e., the signal subspace. $U_{n}$ is the subspace of the eigenvectors corresponding to the 3−k smaller eigenvalues of R, i.e., the noise subspace. $\Sigma_{s}$ and $\Sigma_{n}$ are the diagonal matrix of k larger eigenvalues and 3−k smaller eigenvalues, respectively.

Due to the orthogonality between the steering vector and the noise subspace, the MUSIC spatial spectrum of a single vector hydrophone is obtained as:

(17) $$P_{Music}\left(\theta \right)=\frac{1}{a^{H}\left(\theta \right)U_{n}U_{n}{}^{H}a\left(\theta \right)}$$

The azimuth estimation of the signal can be obtained by scanning the peaks of the spatial spectrum.

### 2.6. Data Fusion Orientation Algorithm Based on Weighted Histogram Statistics

**Figure 5:**
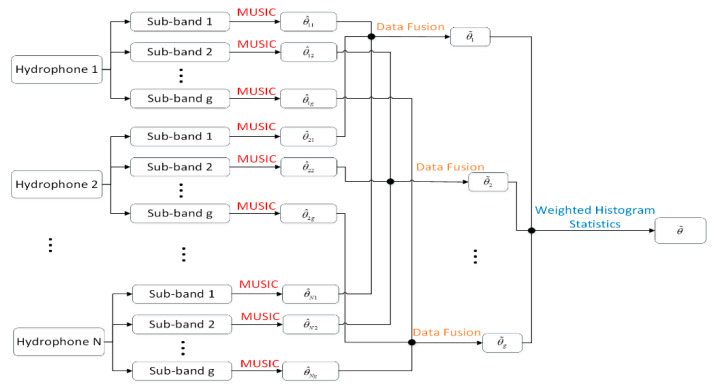
Flow diagram of the data fusion orientation method based on the weighted histogram statistics (DF-WHS).

As mentioned previously, it is hard to achieve better azimuth estimation results than those of a single vector hydrophone when existing array signal processing algorithms are applied to the vector sensor vertical array. To take full advantage of the multiple hydrophones of the vector array, we have derived a method based on data fusion technology to effectively improve the azimuth estimation performance. The presented algorithm contains four steps, and the corresponding flow diagram is shown in Figure 5. 

Firstly, the processing frequency band is divided into several sub-bands that do not coincide with each other. 

Secondly, the high-resolution MUSIC algorithm is applied to estimate the sub-band azimuths for each vector hydrophone. 

Thirdly, the estimation results of each element are fused by the data fusion technique. 

Finally, a series of sub-band fusion results are synthesized in the frequency domain by the weighted histogram method. The angle corresponding to the point with the maximum statistical value is the ultimate target azimuth angle. 

Here, we suppose a vector hydrophone vertical array is composed of N elements, and we adopt the array to collect acoustic signals radiating from the source in the far-field. The processing frequency band B is divided into g non-overlapping sub-bands. Firstly, we adopt the MUSIC high-resolution method to acquire the azimuths of each sub-band. Regarding ${\hat{\theta }}_{ij}$ as the estimated azimuth of the i*-*th vector hydrophone at the j*-*th sub-band, the estimation angle vector of the j*-*th sub-band can be written as:

(18) $${\tilde{\theta }}_{j}={\left[{\hat{\theta }}_{1j},{\hat{\theta }}_{2j},\cdots ,{\hat{\theta }}_{Nj}\right]}^{T},j=1,2,\cdots ,g$$

The results of different hydrophones are fused to obtain a more precise target position. According to the linear fusion model, the estimated azimuths of each sub-band can be expressed as:

(19) $${\tilde{\theta }}_{j}=Hx_{j}+e_{j},j=1,2,\cdots ,g$$

where $x_{j}$ denotes the estimand. $e_{j}={\left[e_{1j},e_{2j},\cdots e_{Nj}\right]}^{T}$ represents the N×1 dimensional measurement error and satisfies the Gaussian distribution, namely, $E\left(e_{ij}=0\right)$. H is the N×1 dimensional mapping matrix between ${\tilde{\theta }}_{j}$ and $x_{j}$. Generally, elements of H are all one due to the same type of each hydrophone, namely $H={\left[1,1,\cdots 1\right]}^{T}$.

However, the estimation results of each element have different accuracy in practice. Thus, we employ the weighted least squares (WLS) method to integrate the data and eliminate the uncertainty. For the high-accuracy hydrophone, the weight setting is larger, but for the low-accuracy hydrophone, the weight is smaller. Hence, the estimation result becomes more accurate after using the WLS method.

The weighted coefficient of N vector hydrophones at the j-th sub-band is written as $w_{j}=\left[w_{1j},w_{2j},\cdots ,w_{Nj}\right]$, which satisfies the relationship $\sum_{i=1}^{N}w_{ij}=1,0\leq w_{ij}\leq 1$. The aim of data fusion is to design appropriate $w_{ij}$ so that the new estimator $w_{j}{}^{T}{\tilde{\theta }}_{j}$ is unbiased to $x_{j}$, that is, $E\left(w_{j}{}^{T}{\tilde{\theta }}_{j}-x_{j}\right)=0$. Under the condition of $E\left(e_{ij}=0\right)$ and $H={\left[1,1,\cdots 1\right]}^{T}$, the equation above holds. In addition, the estimation error is less than the variance of estimation results of any single vector hydrophone. The sum of the square of weighted errors can be expressed as:

(20) $$J_{w}\left({\hat{x}}_{j}\right)={\left[{\tilde{\theta }}_{j}-H{\hat{x}}_{j}\right]}^{T}W_{j}\left[{\tilde{\theta }}_{j}-H{\hat{x}}_{j}\right]$$

where $W_{j}=diag\left(w_{1j},w_{2j},\cdots ,w_{Nj}\right)$. According to the WLS rule, the optimal weight $W_{j}$ is obtained when $J_{w}\left({\hat{x}}_{j}\right)$ reaches the minimum.

Based on the method of finding the minimum, we take the partial derivative of Equation (20) and set it to zero, namely:

(21) $$\frac{\partial J_{w}\left({\hat{x}}_{j}\right)}{\partial {\hat{x}}_{j}}=-H^{T}\left(W_{j}+W_{j}{}^{T}\right)\left({\tilde{\theta }}_{j}-H{\hat{x}}_{j}\right)=0$$

According to the theory mentioned above, the estimated azimuth for the j-th sub-band signal after fusion processing can be written as:

(22) $${\hat{x}}_{j}={\left(H^{T}W_{j}H\right)}^{-1}H^{T}W_{j}{\tilde{\theta }}_{j}=\frac{\sum_{i=1}^{N}w_{ij}{\hat{\theta }}_{ij}}{\sum_{i=1}^{N}w_{ij}}\;j=1,2\cdots ,g$$

Further, the corresponding fusion variance is expressed as:

(23) $$\sigma_{j}{}^{2}=E\left[{\left(x_{j}-{\hat{x}}_{j}\right)}^{2}\right]=\sum_{i=1}^{N}w_{ij}{}^{2}\sigma_{ij}{}^{2}\;j=1,2\cdots ,g$$

where $\sigma_{ij}{}^{2}$ is the variance of the estimated azimuth of the i-th vector sensor in the j-th sub-band. According to the extreme value theory, when the fusion variance reaches the minimum, the weight can be deduced as:

(24) $$w_{ij}=\frac{1/\sigma_{ij}{}^{2}}{\sum_{r=1}^{N}\frac{1}{\sigma_{rj}{}^{2}}\;}\;j=1,2\cdots ,g$$

Substituting Equation (24) into Equation (23), the fusion variance is:

(25) $$\sigma_{j}{}^{2}=\frac{1}{\sum_{i=1}^{N}\frac{1}{\sigma_{ij}{}^{2}}\;}\;j=1,2\cdots ,g$$

From Equation (25), it is indicated that the variance after fusion is less than that of any single vector sensor. To obtain the final result, the estimated azimuths for multiple sub-bands are synthesized in the frequency domain. Generally, the signal energy radiating from a target such as a ship is not uniformly distributed in the frequency domain. The azimuth estimation accuracy of noise-dominated sub-bands is lower. To suppress the influence of noise, we propose a weighted histogram statistic method. The estimated variance of the j-th sub-band signal after fusion processing can be expressed as:

(26) $$\sigma^{\prime }{{}_{j}}^{2}=E\left[{\left({\tilde{\theta }}_{j}-E\left({\tilde{\theta }}_{j}\right)\right)}^{2}\right]\;j=1,2\cdots ,g$$

From Equation (26), we find that if the j-th sub-band is dominated by noise, the estimation results for each element deviate greatly. Thus, $\sigma^{\prime }{{}_{j}}^{2}$ is larger and the corresponding weight should be smaller. On the contrary, the corresponding weight is larger for those sub-bands with low noise. Referring to Equation (24), we define the statistical weight $w_{j}{}^{\prime }$ that satisfies the equation as:

(27) $$w_{j}{}^{\prime }=\frac{1/\sigma^{\prime }{{}_{j}}^{2}}{\sum_{j=1}^{g}\frac{1}{\sigma^{\prime }{{}_{j}}^{2}}\;}\;j=1,2\cdots ,g$$

The conventional uniform weighting method assigns the same weight 1/g to the azimuth estimation result of each sub-band, so it cannot suppress the influence of the sub-band dominated by noise. The weighting processing proposed above adjusts the corresponding weight according to the mean square error (MSE) of each sub-band to reduce the proportion of the results of the noise sub-band. Therefore, the influence of inaccurate estimation results caused by noise is effectively suppressed, and the final estimation result is closer to the true azimuth.

With the designed weight above, the estimated azimuths for multiple sub-bands are synthesized in the frequency domain by adopting the weighted histogram statistics method. The method can be described as:

(28) $$\{\begin{array}{c} n=\left[{\hat{x}}_{j}\cdot 180/\pi \right]\\ \phi \left(n\right)=\phi \left(n\right)+w_{j}{}^{\prime } \end{array}\;j=1,2\cdots ,g$$

where [ ] is the rounding operator. n represents the integer azimuth for each sub-band expressed in the form of an angle. ϕ(n) is the frequency of each integer angle. The angle corresponding to the maximum frequency is the azimuth of the target signal.

It should be noted that the algorithm proposed in this paper is suitable for both optical fiber vector hydrophones and piezoelectric vector hydrophones.

The DF-WHS algorithm takes full advantage of the multiple vector hydrophones and employs the weighted histogram statistics method to restrain the influence of noise sub-bands effectively. Hence, high-precision DOA results can be derived under high and low SNR conditions.

## 3. Simulation

In this section, a performance comparison between the DF-WHS method and several conventional methods is carried out under different SNR conditions. One condition is that the SNRs of all sub-bands are high, and the other is that the noise-dominated sub-bands exist.

### 3.1. High SNR Condition

We first consider the situation where the SNRs for all of the processing frequency band are high. We suppose that a target sound source exists in the far field and radiates a broadband chirp signal with a frequency from 200 to 500 Hz. The azimuth angle of the source is moving from $\theta \;=\;90^{o}$ to $\theta \;=\;270^{o}$ continuously by changing $1^{o}$ per 1 s. The noise satisfies Gaussian and white noise models, of which the frequency band ranges from 100 to 1000 Hz. The processing band is also 100–1000 Hz. We divide the processing frequency band into nine segments, in which each segment has a 100 Hz bandwidth with an SNR of 10dB. A 16-element vector hydrophone vertical array is utilized to collect the signals, and the sampling rate is 12,000 Hz.

**Figure 6:**
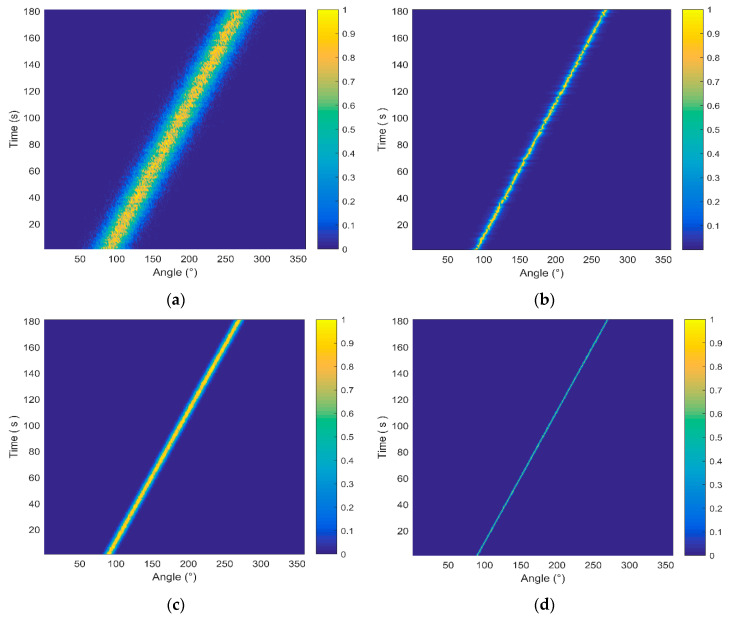
The estimation azimuth results of four methods under high SNR conditions. (**a**) Cross-spectral sound intensity method of single vector hydrophone (CSSI-SVH); (**b**) multiple signal classification (MUSIC) method of single vector hydrophone (M-SVH); (**c**) data fusion algorithm for the vector hydrophone vertical array based on the cross-spectral sound intensity method (DF-CSSI); (**d**) the data fusion algorithm based on the weighted histogram statistics (DF-WHS).

To compare the DOA estimation performance, we discuss the orientation performance of the proposed DF-WHS algorithm and other three conventional algorithms, namely, the cross-spectral sound intensity method of the single vector hydrophone (CSSI-SVH), the MUSIC method of the single vector hydrophone (M-SVH) and the data fusion algorithm for the vector hydrophone vertical array based on the cross-spectral sound intensity technology (DF-CSSI). The corresponding azimuth estimation results are shown in Figure 6.

It is manifested that the beam width in Figure 6b is evidently narrower than that in Figure 6a. In other words, the azimuth estimation performance of the high-resolution MUSIC orientation algorithm is superior to the cross-spectral sound intensity method for a single vector hydrophone. After data fusion processing, the DOA estimation results of the 16-element array in Figure 6c, d are significantly improved compared to those of the single element shown in Figure 6a,b.

The data fusion algorithm takes full use of the output signals of multiple elements and achieves better orientation results than that of a single element. Furthermore, the DF-WHS algorithm has the best performance in high SNR conditions due to the high-resolution MUSIC orientation algorithm used in each sub-band.

### 3.2. Influence of the Noise-Dominated Sub-Bands

To evaluate the orientation performance of the DF-WHS algorithm under the low SNR condition, the noise-dominated sub-bands are considered. The noise frequency band from 100 to 1000 Hz is divided into nine segments with the bandwidth of 100 Hz. The SNRs of each sub-band are set to be −5, 2, 2, 2, −5, −5, −5, −5, and −5 dB, respectively. That is, the signal energy is mainly concentrated in the frequency band of 200–500 Hz.

**Figure 7:**
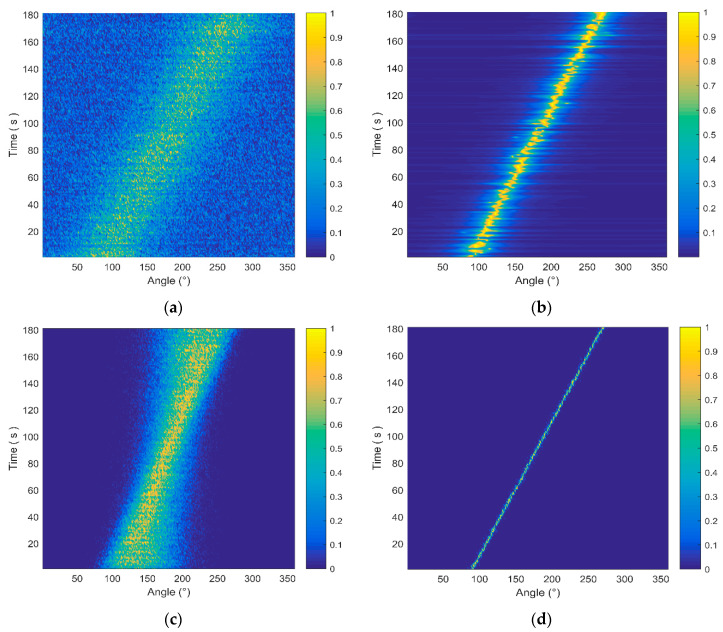
The estimation azimuth results of four methods with the processing frequency band of 100–1000 Hz. (**a**) Cross-spectral sound intensity method of single vector hydrophone (CSSI-SVH); (**b**) MUSIC method of single vector hydrophone (M-SVH); (**c**) data fusion algorithm for the vector hydrophone vertical array based on the cross-spectral sound intensity method (DF-CSSI); (**d**) the DF-WHS algorithm.

In practice, the energy distributions of the signal received are not uniform in the frequency domain, and the noise-dominated sub-bands usually have a crucial influence on the orientation performance. Aiming at verifying the DOA performance of the DF-WHS method, we assume that the processing frequency band is 100–1000 Hz, including six noise-dominated sub-bands. The source is moving from $\theta \;=\;90^{o}$ to $\theta \;=\;270^{o}$ continuously by changing $1^{o}$ per 1 s. The azimuth estimation results corresponding to these four methods are presented in Figure 7. From Figure 7, we can see that the CSSI-SVH, M-SVH, and DF-CSSI methods show a rapid performance degradation because more noise is introduced in the data processing. Taking the DF-CSSI method as an example shows that the beam width (shown in Figure 7c) is still broad even though the data fusion method is adopted, because the estimated azimuths of noise-dominated sub-bands are inaccurate and there is no weight difference for those noise-induced estimation results in the histogram statistics processing. However, we adopt the weighted histogram statistics method in the DF-WHS algorithm, so that these estimation results caused by noise only account for a small proportion of the final statistics. Hence, the DF-WHS algorithm suppresses noise sufficiently and results in a performance improvement in comparison with other three methods mentioned above, particularly under the low SNR condition.

To compare the orientation performance of the methods mentioned above, we calculate the MSE and the correct probability of the azimuth estimation results under different SNR conditions. The Monte Carlo method was carried out 500 times in this simulation. The MSE represents the deviation between the estimated value and the actual value, and the correct probability is defined as the ratio between the number of correct estimation results and the number of observations. Here the estimation result is considered to be correct when the absolute value of the estimated error is less than 1.

**Figure 8:**
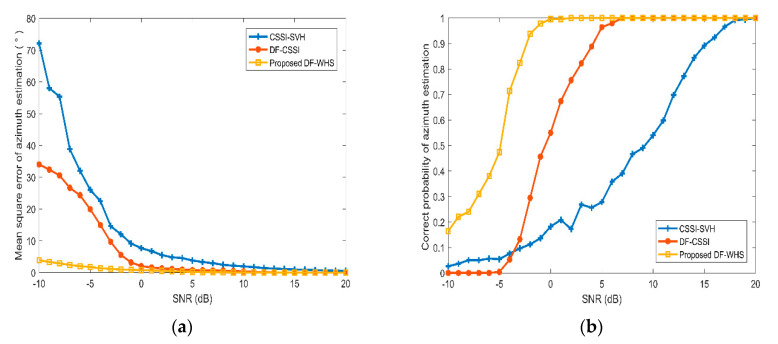
Performance analysis of algorithms with signal-to-noise ratio (SNR) variation. (**a**) Mean square error; (**b**) correct probabilities.

Here we suppose there is a fixed-point target locating in the far field and its azimuth angle is $140^{o}$. The frequency band of the signal and noise are 200–500 Hz and 100–1000 Hz, respectively. The sub-band width is set to be 100 Hz, and the SNR varies from −10 to 20 dB with an interval of 0.5 dB. The processing frequency band is chosen as 100–1000 Hz. Figure 8 shows the estimated MSE and correct probabilities corresponding to CSSI-SVH, DF-CSSI, and DF-WHS algorithms.

From Figure 8, we find that for the three algorithms mentioned above, the MSE decreases and the correct probability increases as SNR increases. In contrast with the CSSI-SVH and DF-CSSI algorithms, the DF-WHS algorithm in this paper still shows the best orientation performance. As shown in Table 1, the estimated MSE of the DF-WHS method is only 3.9° when the SNR is as low as −10 dB. However, the corresponding MSEs of the CSSI-SVH and DF-CSSI algorithms are 72.1° and 34.0° under the same SNR. Furthermore, the minimum SNR required for the DF-WHS algorithm is 0 dB when the correct probability reaches 1, compared to 7 and 20 dB for the CSSI-SVH and DF-CSSI algorithms, respectively. As shown in Figure 8b, the correct probability of the estimated azimuth obtained by the DF-CSSI method is lower than that by the CSSI-SVH method when the SNR varies from −10 to −3 dB. This is because the accuracy of the estimated azimuth acquired by the CSSI-SVH algorithm is already poor under lower SNR. After data fusion processing, the correct probability is not improved but made worse. It is concluded that the DF-WHS algorithm can still provide good performance for azimuth estimation under the condition of low SNR.

## 4. Experimental Analysis

### 4.1. Experiment Introduction

**Figure 9:**
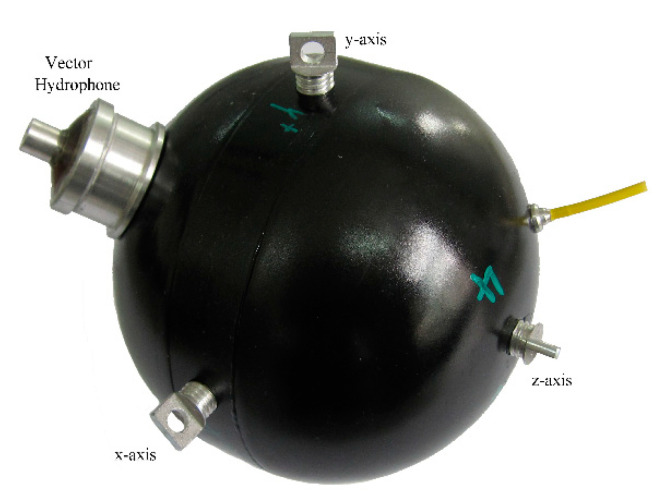
Picture of vector hydrophone.

**Figure 10:**
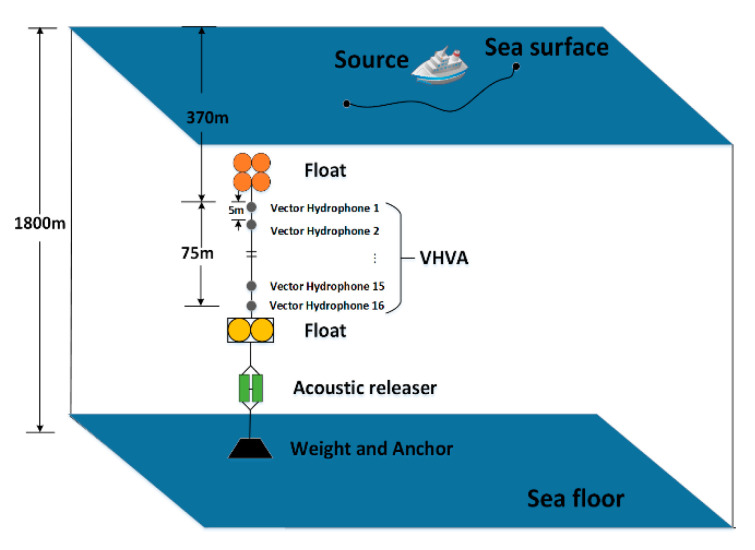
Experiment configuration.

We conducted a sea trial in the South China Sea in 2016 and adopted a 16-element vector hydrophone vertical array. The structure of the vector hydrophone used is presented in Figure 1, and a picture of it is shown in Figure 9. The vertical array was a series of such vector sensors connected in the different depths of the vertical direction, as shown in Figure 10. A moving vessel was chosen as the acoustic source, and the vector sensor array received the broadband radiated noise of the ship. Each element was equipped with an electronic compass to record the three-dimensional underwater attitude of each one. Other experimental parameters were as follows.

**Figure 11:**
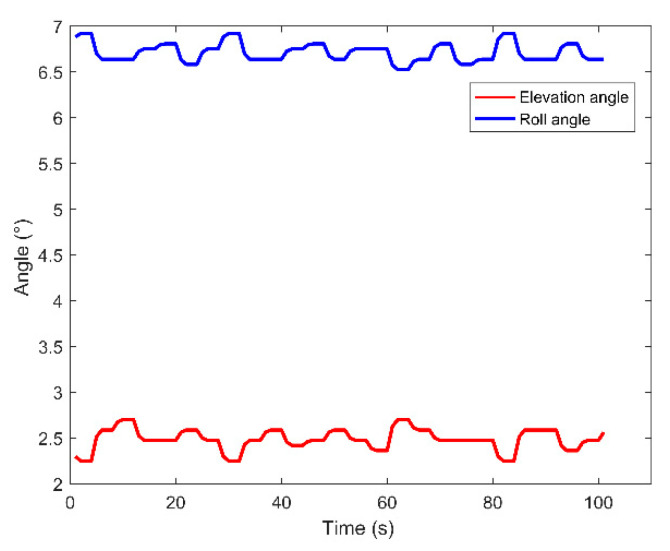
The tilt degree of elevation angle and roll angle during the 100 s processing time.

**Figure 12:**
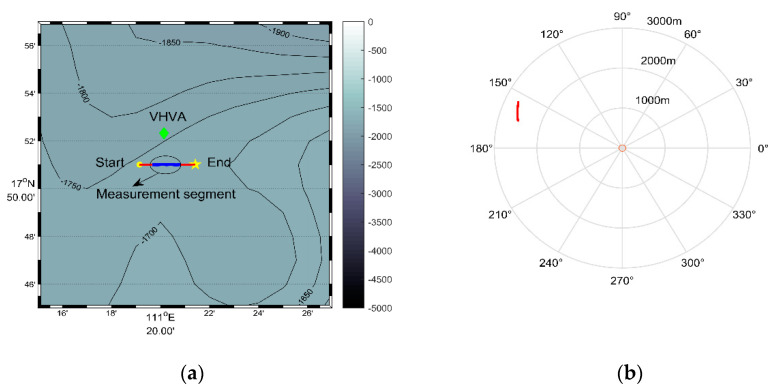
Information obtained by GPS data. (**a**) Ship trajectory map (the green diamond represents the location of vector hydrophone vertical array; the yellow circle and the five-pointed star stand for the starting and ending positions of the trace, respectively; and the solid red line is the trajectory and the solid blue line in the middle is that of the intercepted measurement segment); (**b**) azimuth change obtained by GPS data (the solid red line represents azimuth change during the intercepted measurement segment).

**Figure 13:**
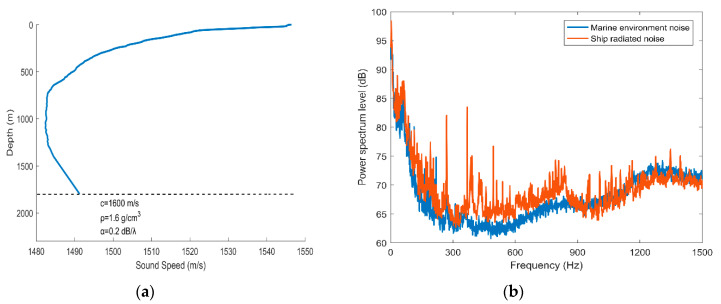
(**a**) Measured sound speed profile; (**b**) spectrum level of the radiated noise from the ship.

- The vector hydrophone vertical array was composed of 16 elements with an interval of 5 m. The top hydrophone was placed at a depth of 370 m, and the bottom hydrophone was at about 445 m. The water depth of the experimental area was about 1800 m.
- The vector hydrophone is composed of a pressure hydrophone and a three-dimensional accelerometer, which collect the pressure and acceleration of ship noise, respectively. Then, the acceleration is converted into velocity by integration.
- The vector hydrophone had a sound pressure sensitivity of –144.3 dB per rad/μPa and acceleration sensitivity of 32.6 dB per rad /g.
- A 100 s signal was chosen from the whole measurement as the processing data. The array tilted due to the disturbance of ocean currents. The tilt degree of the elevation angle and roll angle during the 100 s processing time is presented in Figure 11. The attitude of each vector hydrophone was modified according to the electronic compass data when processing the received data.
- Both the ship and the array were equipped with Global Positioning System (GPS) modules to record real-time spatial positions, as shown in Figure 12a. The azimuth acquired by GPS data during the 100 s processing time is shown in Figure 12b.
- The sound velocity profiler (SVP) was used to measure the sound speed profile (SSP) at the beginning of the experiments. The measured SSP is shown in Figure 13a.
- The sampling rate was 16 kHz. The sea condition was level 3, and no other vessels passed during the test time.

The data collected by each element in different depths were processed to obtain the azimuth information of the sound source. Then, the multiple hydrophones data fusion method was applied to achieve a higher-precision real-time target azimuth.

Figure 13b shows the spectrum curves of the radiated noise received from the ship and the collected marine environmental noise. At this time, the ship was about 2.5 km from the array. It is indicated that the energy of the ship noise is mainly concentrated on the frequency band of 200–900 Hz, where the ship noise energy is about 3 dB higher than that of the marine environment noise. Due to the uneven distribution of ship noise energy in the frequency domain, two processing frequency bands were considered. One was 200–900 Hz and the other was 100–1500 Hz. The CSSI-SVH method, DF-CSSI method, and DF-WHS algorithm were adopted to process the 100-s signal intercepted from the sampling data.

### 4.2. Processing Frequency Band of 200–900 Hz

**Figure 14:**
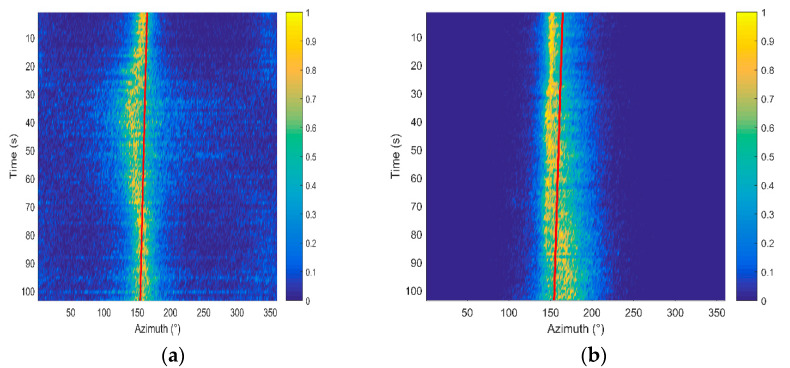

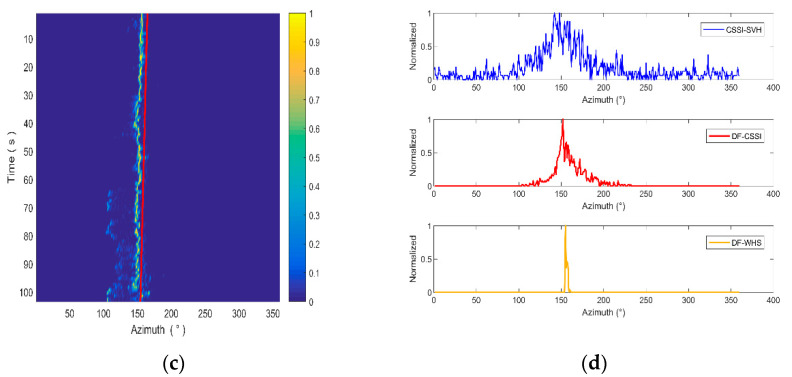
Results of processing frequency band of 200–900 Hz. (**a**) Cross-spectral sound intensity method of single vector hydrophone (CSSI-SVH); (**b**) data fusion algorithm for the vector hydrophone vertical array based on the cross-spectral sound intensity method (DF-CSSI); (**c**) the DF-WHS algorithm; (**d**) the beam pattern of the three methods.

In this case, the processing frequency band chosen is consistent with the energy distribution of the ship noise in the frequency domain. The sub-band has a width of 10 Hz and the azimuth estimation results of these three methods are presented in Figure 14.

In Figure 14a–c, the solid red lines represent the real azimuth angles of the ship calculated through GPS data. During the sampling time, the azimuth of the moving ship changes from 165° to 155°. From Figure 14a, we can see that the azimuth estimation results of the CSSI-SVH method are distributed from 135° to 170°, and the exact azimuth is hard to identify due to the high side lobe. In the time range of 20 to 70 s, the beam width is widened and drifts away from the real azimuth angle. After data fusion processing, the statistics values in Figure 14b are more concentrated near the true value, while the side lobe is still high and the beam width is broad. When employing the DF-WHS algorithm, the beam width is significantly narrower and is maintained at about 4° during most of the sampling time. As shown in Figure 14c, the estimated azimuth is basically consistent with the results calculated by GPS data. Figure 14d shows the beam patterns of the three algorithms at the same time. The results reveal that the DF-WHS method in this paper has the narrowest beam width and the lowest side lobe, and thus has the best DOA estimation performance.

The corresponding beam widths of the three algorithms are listed in Table 2.

### 4.3. Processing Frequency Band of 100–1500 Hz

**Figure 15:**
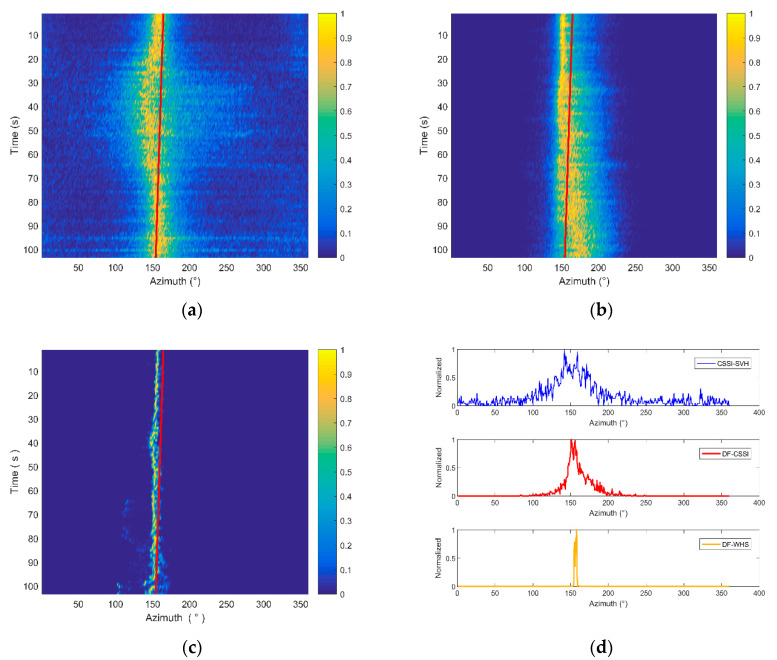
Results of the processing band of 100–1500 Hz. (**a**) Cross-spectral sound intensity method of single vector hydrophone (CSSI-SVH); (**b**) data fusion algorithm for the vector hydrophone vertical array based on the cross-spectral sound intensity method (DF-CSSI); (**c**) the DF-WHS algorithm; (**d**) the beam pattern of the three methods.

Under this situation, the processing frequency band is chosen as 100–1500 Hz, which is evidently wider than the energy distribution band of the ship noise. The sub-band is also divided with an interval of 10 Hz. The azimuth estimation results of these three methods and corresponding beam patterns are presented in Figure 15.

Compared with the estimation results presented in Figure 14, the beam width is much broader, and the peak energy is more dispersed, when using the CSSI-SVH and DF-CSSI methods because more noise is introduced when the processing frequency band is much broader than that of the signal. However, the estimation result of the DF-WHS algorithm is less affected and can distinguish the angle more clearly. Furthermore, its side lobe is significantly lower, and the beam width is significantly narrower. The corresponding beam widths acquired by the three methods are listed in Table 3. As shown in Figure 15d, the bimodal structure appears in the beam patterns when adopting the CSSI-SVH and DF-CSSI methods. It is difficult to accurately determine the estimated azimuths of the target due to the similar peak energy. For the DF-WHS method, the estimated azimuth is more accurate and in good agreement with the angles calculated by GPS data due to the effective noise suppression in the processing. It is concluded that the DF-WHS algorithm showed better orientation performance in the sea trial, especially under the condition of low SNR.

In this section, we carried out a sea trial to verify the performance of the DF-WHS method. In contrast to the traditional CSSI-SVH and DF-CSSI methods, the azimuth obtained by the DF-WHS algorithm is more accurate, the beam width is significantly narrower, and the side lobes are significantly lower. It has been proved that the DF-WHS algorithm can provide superior orientation performance and is little affected by noise. There are some suggestions when using this algorithm.

1. The SNR of the signal received cannot be too low, and most hydrophone elements of the array need to have a good orientation result.
   The music high-resolution algorithm is greatly affected by SNR. As shown in Figure 8b, when the SNR is less than –4 dB, the correct probability for the DF-WHS method is less than 0.7. Especially when the SNR is –10 dB, the correct probability is as low as 0.16 (the yellow line), because in the case of a low SNR of each sub-band, the orientation performances of all elements are poor. The fusion result of the DF-WHS method is also very poor. Therefore, this method depends on the element orientation result, and most hydrophone elements of the array need to have a good orientation result.
2. This method is more suitable for a moving target with a low azimuth change rate.
   We applied the approach of segmentation in this paper, and the time length of each segment was set to be 1s. If the azimuth of the moving target θ changes quickly, the estimated azimuth within 1 s is much closer to the intermediate value of the azimuth change range, and the correct probability will decrease accordingly. Therefore, the moving speed of the target is suggested to be low enough, or the target is far from the vertical array. According to the proposed algorithm, we aspire for the azimuth change rate of the target △θ to be less than 1°.
3. The target and array should avoid being located in the shadow zone.
   In the shadow zone, the sound field is mainly dominated by bottom-reflected rays rather than direct rays. The arrival angle does not truly reflect the target azimuth angle. Thus, the orientation performance may be inaccurate.

## 5. Conclusions

In this paper, we propose a data fusion orientation algorithm based on the weighted histogram statistics method to obtain more accurate DOA estimation results for the vector hydrophone vertical array. To verify its significant improvement in terms of orientation performance, we compare the DF-WHS algorithm and other traditional azimuth estimation methods through simulation and a sea trial. Both the simulation and experiment results indicate that the proposed DF-WHS method performs better than other algorithms, especially in a high noise environment. The high-resolution MUSIC algorithm is adopted to significantly improve the accuracy, and the weighted histogram statistics method is designed to effectively suppress the noise. Therefore, the beam width of the estimated azimuths for the DF-WHS method is significantly narrower, the side lobes are significantly lower, and the mean square error is considerably smaller. Finally, we analyze the limitations of the algorithm and give some suggestions for its application. The DF-WHS method provides new ideas for high-resolution detection in deep oceans based on the vector hydrophone vertical array.

## Figures and Tables

**Table 1:** Important angles and SNRs.

Algorithm Name	Estimated MSE When SNR = −10 dB	Minimum SNR Required When the Correct Probability Reaches 1
CSSI-SVH	72.1°	20 dB
DF-CSSI	34.0°	7 dB
DF-WHS	3.9°	0 dB

**Table 2:** Beam widths of the three algorithms in the 200–900 Hz processing frequency band.

Algorithm Name	Beam Width
CSSI-SVH	35°
DF-CSSI	25°
DF-WHS	4°

**Table 3:** Beam widths of the three algorithms in the 100–1500 Hz processing frequency band.

Algorithm Name	Beam Width
CSSI-SVH	40°
DF-CSSI	35°
DF-WHS	6°
